# PARENT-CHILD RELATIONSHIPS AND SLEEP QUALITY AMONG CHINESE AGING PARENTS

**DOI:** 10.1093/geroni/igz038.2486

**Published:** 2019-11-08

**Authors:** Haowei Wang, Kyungmin Kim, Jeffrey Burr, Bei Wu

**Affiliations:** 1 University of Massachusetts Boston, Boston, Massachusetts, United States; 2 New York University, New York, New York, United States

## Abstract

Relationships with adult children play an important role in older adults’ well-being. However, little is known about the association between parent-child relations and aging parents’ sleep quality, which is an emerging health issue that is closely related to individuals’ physical and mental well-being in later life. With the largest aging population, China has experienced rapid changes of family structure and traditional norms regarding parent-child ties. This study focused on different dimensions of parent-child relationships (i.e., upward/downward financial and instrumental support, emotional closeness) in Chinese aging families. This study examined the association between parent-child relationships and older parents’ sleep quality, comparing one-child and multiple-children Chinese families. Utilizing the 2014 wave of the Chinese Longitudinal Aging and Social Survey, we analyze data from 8,450 respondents (aged 60+) who had at least one living child. Descriptive analysis showed that parents with multiple children engaged in more intense financial exchanges, less frequent instrumental support, and lower levels of emotional closeness with their adult children compared to their counterparts with only one child. Logistic regression models revealed that older parents who received more instrumental support were more likely to report sleep difficulty in both one-child and multiple-children families. For parents with multiple children, the overall level of financial transfers from children was negatively associated with having sleep difficulties, while the variability of financial transfers across multiple children was positively associated with having sleep difficulty. Findings highlight the importance of considering family dynamics in studying sleep quality among Chinese older adults.

